# Longitudinal Study of Patients with Connective Tissue Disease–Interstitial Lung Disease and Response to Mycophenolate Mofetil and Rituximab †

**DOI:** 10.3390/diagnostics14232702

**Published:** 2024-11-30

**Authors:** Yan Li, Sehreen Mumtaz, Hassan Z. Baig, Isabel Mira-Avendano, Benjamin Wang, Carlos A. Rojas, Justin T. Stowell, Elizabeth R. Lesser, Shalmali R. Borkar, Vikas Majithia, Andy Abril

**Affiliations:** 1 Division of Rheumatology, Mayo Clinic, Jacksonville, FL 32224, USA; liyan.residency@gmail.com (Y.L.); mumtaz.sehreen@mayo.edu (S.M.); majithia.vikas@mayo.edu (V.M.); 2 Division of Pulmonary, Allergy and Sleep Medicine, Mayo Clinic, Jacksonville, FL 32224, USAisabel.c.miraavendano@uth.tmc.edu (I.M.-A.); 3 Department of Critical Care Medicine, Mayo Clinic, Jacksonville, FL 32224, USA; 4 Division of Thoracic, Cardiac Radiology, Mayo Clinic, Jacksonville, FL 32224, USA; rojas.carlos@mayo.edu (C.A.R.);; 5 Biostatistics Unit, Mayo Clinic, Jacksonville, FL 32224, USA; rhodes.elizabeth@mayo.edu (E.R.L.); borkar.shalmali@mayo.edu (S.R.B.)

**Keywords:** immunosuppressive agents, connective tissue diseases, interstitial lung disease, respiratory function tests

## Abstract

**Background/Objective:** To investigate the effect of mycophenolate mofetil (MMF) and rituximab (RTX) on pulmonary function test (PFT) results in a mixed cohort of patients with connective tissue disease-associated interstitial lung disease (CTD-ILD), longitudinally followed up for 1 year in a single academic center. **Methods:** Patients with CTD-ILD were identified in electronic medical records from 1 January 2009 to 30 April 2019. Prescribed MMF and RTX doses, dosage changes, and therapy plans were analyzed individually with improvement in PFT outcomes determined using multivariable linear regression models during 12-month follow-up. **Results:** Forty-seven patients with CTD-ILD, treated with MMF, RTX, or both, were included. Patients on combined MMF and RTX had worse PFT outcomes at baseline compared with patients on monotherapy. Substantial improvement was observed among all PFT outcomes from baseline to 12 months, regardless of medication dosage or therapy plans. The diffusing capacity of the lungs for carbon monoxide (DLCO) worsened by an average of 7.21 mL/(min*mmHg) (95% CI, 4.08–10.33; *p* < 0.001) among patients on RTX compared to combined therapy. Patients on higher doses of MMF at baseline experienced an average increase of 0.93 (95% CI, 0.04–1.82) units in DLCO from baseline to 6 months (*p* = 0.04) and a 2.79% (95% CI, 0.61–4.97%) increase in DLCO from 6 to 12 months (*p* = 0.02) within patients on concurrent RTX at 6-month follow-up. **Conclusions:** The treatment of CTD-ILD with MMF and/or RTX was associated with overall improvement in PFT outcomes. Combined therapy resulted in significant improvements in DLCO compared with monotherapy. Higher doses of MMF also provided greater improvements in DLCO.

## 1. Introduction

Interstitial lung diseases (ILDs) are a group of diffuse lung parenchymal infiltrative diseases with various etiologies and distinct clinical features. Connective tissue diseases (CTDs) are a commonly identified cause for ILD that result in immune-mediated tissue injury involving the lungs [1]. In patients with CTDs, the presence of ILD is associated with substantial morbidity and mortality. For example, the 5-year mortality rate is threefold higher in patients with rheumatoid arthritis and systemic sclerosis complicated by ILD [2,3]. However, compared with idiopathic interstitial pneumonias, patients with CTD-associated ILD (CTD-ILD) are more likely to respond to immunosuppressive therapy and have a better prognosis [4].

The concept of interstitial pneumonia with autoimmune features (IPAF) was proposed by the European Respiratory Society and American Thoracic Society in 2015 [5]. It identifies a unique group of patients with ILD who present with autoimmune features without fulfilling the classification criteria for any definitive CTD. The management of CTD-ILD and IPAF involves immunosuppression with various agents, such as corticosteroids, azathioprine, and mycophenolate mofetil (MMF). These have shown promising results in small cohorts of patients, improving or stabilizing pulmonary function test (PFT) results over time, although randomized controlled trials are needed to further address this question [6].

Cyclophosphamide has been used for the initial approach to patients with corticosteroid-refractory severe CTD-ILD [7]. However, its substantial adverse effects profile has largely limited its use. Azathioprine has been widely used as a maintenance corticosteroid-sparing agent, although efficacy data from large clinical trials are lacking. In the past decade, MMF has gained popularity as both an initial and maintenance therapy in CTD-ILD [8]. Fischer et al. [9] have found MMF to be an effective agent for a wide spectrum of CTD-ILDs. More recently, rituximab (RTX) has demonstrated benefits in various CTD-ILDs [10,11], including severe cases [12,13]. RTX was demonstrated to improve forced vital capacity (FVC) and quality of life in CTD-ILD patients and was relatively well tolerated [14]. RTX in combination with MMF was superior to MMF alone in patients with ILD [15].

There are ongoing clinical trials to further investigate the efficacy of various immunosuppressive therapies in the treatment of CTD-ILD. To our knowledge, there is a lack of prospective longitudinal data on the use of MMF and RTX in CTD-ILD. This study aims to show the effect of MMF and RTX on PFT outcomes in a mixed cohort of patients with CTD-ILD, including IPAF, longitudinally followed up for 1 year in a single academic center.

## 2. Patients and Methods

### 2.1. Prospective Study Cohort

Patients with CTD-ILD were identified based on electronic medical records from the Mayo Clinic in Jacksonville, Florida, and Rochester, Minnesota, from 1 January 2009 to 30 April 2019. A chart review was conducted on a total of 413 patients prior to 30 April 2018, among whom 275 were excluded due to a final diagnosis of idiopathic pulmonary fibrosis, hypersensitivity pneumonitis, cryptogenic organizing pneumonia (OP), and other unspecified ILDs (Supplemental Figure S1). The diagnosis of a specific CTD was based on assessment by a specialist Rheumatologist. Among 138 patients with confirmed CTD-ILD based on multidisciplinary evaluation, 91 were further excluded if they received a transplant, had mild disease activity on observational follow-up, treatment with agents other than MMF and RTX, or incomplete 12-month follow-up data. Forty-seven patients with CTD-ILD, treated with MMF or RTX and followed for at least 12 months, were included in the cohort (Supplemental Tables S1–S6). Patients were not excluded if they had side effects. Patients who did not have 12 months follow-up data (including office visit and imaging/pulmonary function tests) were excluded.

The baseline visit was defined as either the time of diagnosis of CTD-ILD or initiation of treatment with MMF or RTX, whichever occurred later. The prescribed dose depended on patient and physician consensus at the beginning of each follow-up (baseline and 6 months). The following data were extracted from initial (baseline), 6-month, and 12-month visits: demographics, specific CTD diagnosis, pertinent clinical symptoms, basic laboratory and serology tests, PFT outcomes, chest computed tomography (CT), and treatments. Chest CT studies were independently reviewed by 2 radiologists and classified based on the 2018 ATS guidelines [16]. The images were reviewed blinded to clinical information. The study was approved by the Mayo Clinic Institutional Review Board (IRB number 19-003240 with approval date of 15 April 2019).

### 2.2. Statistical Analysis

The prescribed MMF and RTX doses, changes in dosages, and therapy plans were analyzed individually with PFT improvement using multivariable linear regression models. Dose was defined as a measure of the administered medication amount, whereas therapy plan represented whether the patient was prescribed MMF only (regardless of prescribed dose), RTX only, or a combination of both at baseline and 6-month follow-up. Baseline and 6-month treatment doses of MMF, RTX, and prednisone were modeled with changes in outcomes from baseline to 6 months and 6 months to 12 months, respectively. Secondary multivariable linear regression models were fit to evaluate the association between therapy plan and overall change in PFT outcomes from baseline to 12-month follow-up. Average linear change was estimated for MMF and RTX monotherapy compared to patients on combined or no therapy. Tertiary multivariable linear regression models were fit to understand baseline to 6-month dosage change in MMF, RTX, and prednisone and the possible impact on PFT outcomes from 6 months to 12 months. The average linear change in PFT outcomes was estimated for every 1 g/d increase in MMF, 2 g/d increase in RTX, and 10 mg/d decrease in prednisone for the primary and tertiary models. Positive estimates were indicative of PFT improvement. All models were adjusted for age and sex at baseline except for interaction analyses between MMF, RTX, and prednisone due to the exploratory nature of this study.

Continuous variables were described using the median and range, while categorical variables were summarized using frequency and percent. Changes in PFT outcomes were calculated by subtracting the earliest from the latest follow-up measures (i.e., 6 months − baseline, 12 months − 6 months, and 12 months − baseline). The Kruskal–Wallis test was used to assess continuous differences between therapy plans and whether there was any change in overall PFT outcomes. The Pearson’s χ^2^ test was used to evaluate proportional differences between therapy plans. All tests were 2-sided and *p*-values less than 0.05 were considered statistically significant. Corresponding 95% CIs were estimated. Statistical analyses were performed using R Statistical Software (version 3.6.2; R Foundation for Statistical Computing, Vienna, Austria).

## 3. Results

The baseline characteristics of the 47 patients are summarized in Table 1. The median (range) age was 58 (32–79) years, 34 (72.3%) were women and the majority were White (85.7%). Eighteen patients (38.3%) were former smokers, with none currently smoking. Within the cohort, 19 patients (40.4%) were diagnosed with antisynthetase syndrome, and 7 (14.9%) with IPAF. The MMF dose ranged from 1 to 3 g/d at the discretion of the physician, while the patients prescribed RTX were given 2 g per course. A total of 28 patients (59.6%) were on MMF monotherapy, 6 (12.8%) were on RTX monotherapy, and 13 (27.7%) received combined therapy with MMF and RTX or were switched to RTX because of intolerance or toxicity associated with MMF. All seven patients with IPAF received MMF monotherapy throughout the study period.

### 3.1. Overall PFT Outcomes

A summary of the PFT outcomes at each follow-up period are summarized in Table 2. Overall, significant improvements were observed among all PFT outcomes from baseline to 12 months in this study cohort, regardless of medication doses or therapy plans (*p* ≤ 0.006). All PFT outcomes from baseline to 6 months showed significant improvement, except for diffusing capacity of the lungs for carbon monoxide (DLCO) (*p* = 0.05). In contrast, only DLCO and percentage of DLCO (DLCO%) were observed to improve between the 6-month and 12-month follow-ups (*p* = 0.02 and 0.003, respectively).

### 3.2. PFT Analysis with Combined MMF and RTX Therapy Compared with Monotherapy Plans

Among the 13 patients on combined therapy, 10 (76.9%) received MMF and RTX concomitantly. Of these patients, nine received MMF at baseline, with RTX added at 6 months, and one received MMF combined with RTX from baseline. The other three (23%) patients were switched from MMF to RTX due to lack of response or intolerance to MMF. Seven patients (53.8%) had antisynthetase syndrome, and two (15.4%) had seropositive rheumatoid arthritis (Table 1). Patients on combined therapy had a baseline median (range) forced vital capacity (FVC) of 1.92 (1.20–2.45), percentage of FVC (FVC%) of 50.00% (41.00–65.00%), DLCO of 8.60 (5.80–16.70), and DLCO% of 34.00% (25.00–57.00%), which were lower than with MMF or RTX monotherapy (only significant for FVC%; *p* = 0.005).

No significant differences were observed in terms of FVC and FVC% from baseline to 12 months between therapies (Table 2). However, DLCO was observed to worsen by an average of 7.21 mL/(min*mmHg) (95% CI, 4.08–10.33; *p* < 0.001) for patients receiving RTX monotherapy compared to those on combined therapy.

### 3.3. PFT Analysis with MMF and RTX Doses at Baseline and 6 Months

The relationship between medication doses at baseline and 6 months and their association with PFT outcomes on the subsequent 6-month follow-up are summarized in Table 3, and an interaction analysis is presented in Supplemental Table S1. Throughout the 12-month study period, overall FVC and FVC% changes were not associated with any MMF or RTX dose. However, an average 0.29 (95% CI, 0.09–0.50) unit improvement in FVC from baseline to 6 months was associated with a 1 g/d increase in MMF in patients on concurrent RTX at baseline (*p* = 0.01; Supplemental Table S3).

An association between DLCO and MMF was observed in the first half of the study. Patients on higher doses of MMF at baseline experienced an average increase of 0.93 (95% CI, 0.04–1.82) units in DLCO from baseline to 6 months (*p* = 0.04). In the second half of the study, an average of 2.79% (95% CI, 0.61–4.97%) increase in DLCO% from 6 to 12 months was observed for every 1 g/d increase in MMF in patients on concurrent RTX at 6-month follow-up (*p* = 0.02; Supplemental Table S3). Moreover, patients who were newly prescribed RTX at 6 months were observed to experience a significant improvement in DLCO% (8.06% [95% CI, 2.12–14.00%]; *p* = 0.01; Table 4).

### 3.4. Role of Prednisone

Among all 47 patients in the study cohort, the median (range) prednisone dose was 30 (0–60) mg/d at baseline, 10 (0–60) mg/d at 6 months, and 5 (0–40) mg/d at 12 months. Reducing the dose of prednisone at 6 months did not significantly impact the PFT outcomes at 12 months (Table 4). The interaction analysis of decreasing prednisone dose (per 10 mg/day) and MMF dose with PFTs is depicted in Supplemental Table S2. Overall, there were no major interactions between prednisone, MMF, and RTX doses and their association with PFT outcomes (Supplemental Tables S4 and S5). Further investigation revealed no interaction between decreased prednisone with MMF or RTX dosage changes at 6 months and changes in PFT outcomes from 6 to 12 months (Supplemental Tables S5 and S6).

### 3.5. Association Between CT Findings and PFT Outcomes

The predominant radiographic patterns identified were nonspecific interstitial pneumonia (NSIP) with overlapping organizing pneumonia (OP) in 21 (44.7%) patients, NSIP in 12 (25.5%), and equal cases of usual interstitial pneumonia and OP in 4 (8.5%) each (Table 1). Moreover, 14 of 19 patients with antisynthetase syndrome (73.7%) had NSIP with overlapping OP, 4 (21.1%) had NSIP, and 1 (5.3%) had OP.

From baseline to 6 months, 13 patients (38.2%) showed improvement on CT, while 21 (61.8%) remained stable and none showed progression (13 patients were missing the information). During the first 6 months of therapy, no PFT outcome improvements correlated with imaging improvement (Table 5). From 6 to 12 months, 2 patients (7.4%) improved on CT assessment, while 21 (77.8%) remained stable and 4 (14.8%) worsened (information was missing for 20 patients). Patients who improved on CT had FVC and FVC% increases of 0.29 (95% CI, 0.052–0.56) and 9.25% (95% CI, 0.67–17.83%; *p* = 0.04), respectively. Overall, from baseline to 12-month follow-up, 12 patients (35.3%) improved on imaging, 20 (58.8%) remained stable, and 2 (5.9%) worsened depending on available PFT follow-up (information was missing for 13 patients). No notable association between PFT improvement and imaging improvement was noted, regardless of baseline immunosuppressant use or dose from baseline to 12-month follow-up.

## 4. Discussion

We present an observational study where the longitudinal effect of MMF and RTX in pulmonary physiology was analyzed in 47 patients with different clinical features of CTD-ILD, who were naïve to MMF and RTX prior to enrollment. In our cohort, 28 patients received MMF monotherapy, 6 received RTX monotherapy, and 13 received combined therapy with MMF and RTX (10 patients) or were switched to RTX due to intolerance or toxicity associated with MMF (3 patients). Patients on combined therapy had lower FVC, FVC%, DLCO, and DLCO% at baseline compared with monotherapy groups, indicative of more advanced pulmonary dysfunction. In particular, 7 of 13 patients in the combined therapy group had antisynthetase syndrome, which is known to have more aggressive ILD [17,18,19]. These patients exhibited significant improvement in DLCO when compared to RTX monotherapy. Nine of ten patients on combined therapy were on MMF at baseline, with RTX added at 6 months. An average of 8.06% improvement in DLCO% was observed at 12 months, suggesting that such benefit could be noted even within 6 months in this subgroup of patients.

A higher dose of MMF (1 g/d difference) was noted to be associated with better PFT outcomes in a subgroup analysis. Patients on a higher dose of MMF had significant marginal improvement in DLCO from baseline to 6-month follow-up. Those on concurrent RTX were noted to have significant improvement in FVC from baseline to 6-month and DLCO% from 6 to 12 months.

We have an established Pulmonary–Rheumatology Clinic, where patients undergo standardized multidisciplinary evaluation to define their diagnosis and plan of care. When immunosuppressive therapy is considered, it is our practice to treat patients with MMF as a first choice and RTX in case of no response to MMF or when a remarkable myopathic component is present. This protocol has been established based on published evidence, although it is supported mostly by retrospective data [9,12,13,20,21,22]. Our study also supports this established protocol, and as shown in the summary of PFT outcomes (Table 2), treatment with MMF and/or RTX resulted in significant improvements in all PFT outcomes from baseline to 12-month follow-up.

Based on prior publications, cyclophosphamide was considered an option when the results of the Scleroderma I trial were extrapolated to all CTD-ILDs [23]. In this double-blind, randomized, placebo-controlled clinical trial, a significant, but modest treatment effect over FVC was observed, with the mean absolute difference between the two groups of 1.95% in FVC (95% CI, 1.2–2.6%; *p* < 0.01) favoring cyclophosphamide. However, after 1 year off treatment, no additional benefit was noted (no continuous improvement in lung function was seen), and after 24 months, no significant difference was seen with placebo [24].

Subsequent noncontrolled trials evaluated the effect of MMF in CTD-ILD [20,21,22,25,26], but those trials included no more than 28 patients, and only one had 19 patients with CTD other than scleroderma [22]. In 2013, the largest cohort to date was published [9]. This cohort represented different kinds of CTD: 44 systemic sclerosis, 32 polymyositis or dermatomyositis, 18 rheumatoid arthritis, and 19 lung-dominant CTD. MMF was given for at least 6 months, with an average of 2.5 years. It was found that the medication had good tolerability and was associated with significant improvement or stability of FVC and DLCO over 156 weeks of therapy in all patient groups. However, this was a retrospective cohort, and 50% of the patients were treated with a different immunosuppressive medicine before MMF was started [9].

In our cohort, the median dose of prednisone was 30 mg/d at baseline, with gradual tapering to a median of 5 mg/d by 12-month follow-up, with relatively stable extra-pulmonary manifestations. The reduction of prednisone dosage at 6 months was not associated with changes in PFT outcomes at 12 months, and no significant interaction was found between prednisone, MMF, and RTX doses on PFT outcomes, suggesting that MMF, RTX, or both played a major role in PFT outcomes. This has been demonstrated before, when improvement in PFT outcomes continued during MMF intake, despite decreased prednisone doses [9].

Our study supports the use of MMF in ILD associated with idiopathic inflammatory myopathies, including antisynthetase syndrome, as 40% of our cohort was represented by patients with this diagnosis. So far, the effect of MMF on ILD associated with idiopathic inflammatory myopathies is not clear; two retrospective trials have reported no significant difference between azathioprine and MMF [26,27], while another has demonstrated significant improvement in FVC over time while on MMF [9].

The anti-B cell agent, RTX, was originally used to eradicate CD20^+^ cells in B-cell lymphomas. Later, it was applied for the treatment of autoimmune disorders. Retrospective trials have evaluated the effect of RTX in ILD associated with antisynthetase syndrome, demonstrating significant improvement in FVC and DLCO over a year of treatment; however, they included no more than 25 patients [12,28,29]. RECITAL investigators demonstrated that CTD-ILD patients on RTX had increased FVC, increased quality of life measures, and was well tolerated [14], although superiority to cyclophosphamide was not determined.

There has been a lack of studies directly comparing MMF and RTX for the management of CTD-ILD. More recently, a randomized controlled trial, EVER-ILD, demonstrated the superiority of RTX and MMF in combination compared to MMF alone in ILD with an NSIP pattern. An increase in percent predicted FVC and progression free survival up to 6 months was recorded [15]. These findings are consistent with our study, and in our cohort, significant improvements were observed among all PFT outcomes from baseline to 12 months, regardless of medication doses or therapy plans. A combined therapy and higher dose of MMF were associated with significant improvements in DLCO and DLCO% marginally and within subgroup analyses. Although the sample size is limited in these subgroups, such benefit is of clinical importance and merits further investigation. There was no significant difference in FVC between the medication doses or therapy plans.

Most radiologic patterns in our cohort were NSIP with overlapping OP or NSIP alone, correlating with what has been described in patients with CTD-ILD, and particularly in antisynthetase syndrome [30,31]. At the same time, most cases demonstrated no significant imaging changes from baseline to 12-month follow-up, despite improvement in PFTs with any evaluated therapy, which may be explained by the short follow-up period.

A few limitations need to be taken into consideration when interpreting these results. The primary disadvantage is the sample size of our study. It may have not been adequately powered to detect significant changes associated with treatment doses or therapy plans. Although there were many trending associations (*p* < 0.10) and distinct visual patterns, they were not explicitly reported because the *p*-value did not meet the 0.05 threshold. These associations would benefit from further investigation. The second limitation is the follow-up duration. While the average peak improvement associated with MMF therapy has previously been reported to occur around 18 to 21 months [9,32], our study duration was 12 months. Thus, the full effect of prescribed treatment with MMF or RTX may have been biased. As an observational study without random treatment assignments, another limitation is the potential influence of treatment choice by physicians’ clinical judgment, patients’ preference, disease progression, and access to treatment. The observational nature of our study carries the limitations stemmed from the confounding variables, selection bias and incomplete data. However, patients on combined therapy with MMF and RTX still exhibited a better response in terms of DLCO, despite overall worse PFT at baseline compared with the monotherapy groups.

## 5. Conclusions

In this observational longitudinal study of patients with CTD-ILD, treatment with MMF and RTX was associated with an overall improvement among all PFT outcomes from baseline to 12 months, with combined therapy resulting in significant improvement in DLCO compared with monotherapy, despite patients with more advanced pulmonary dysfunction at baseline. In particular, patients with antisynthetase syndrome exhibited promising responses to combined treatment. Higher doses of MMF also provided more benefit in regard to DLCO. Studies on a larger scale with an extended follow-up are warranted to further investigate the benefit of MMF and RTX in patients with CTD-ILD.

## 6. Key Points

In patients with CTD-ILD, treatment with mycophenolate mofetil and rituximab is associated with PFT improvement. Combined therapy and higher doses of mycophenolate mofetil are likely more beneficial. Patients with antisynthetase syndrome exhibited a promising response to treatment.

## Figures and Tables

**Table 1:** Baseline characteristics stratified by therapy plans.

	Combination (*N* = 13)	Mycophenolate (*N* = 28)	Rituximab (*N* = 6)	Total (*N* = 47)	*p*-Value
Age, years	53 (32, 69)	59 (35, 79)	61 (36, 73)	58 (32, 79)	0.18
Females	10 (76.9%)	22 (78.6%)	2 (33.3%)	34 (72.3%)	0.073
Ethnicity					0.61
White	10 (83.3%)	21 (84.0%)	5 (100.0%)	36 (85.7%)	
Black or African American	2 (16.7%)	2 (8.0%)	0 (0.0%)	4 (9.5%)	
Asian	0 (0.0%)	2 (8.0%)	0 (0.0%)	2 (4.8%)	
Smoker					0.79
Never	7 (53.8%)	18 (64.3%)	4 (66.7%)	29 (61.7%)	
Former	6 (46.2%)	10 (35.7%)	2 (33.3%)	18 (38.3%)	
Clinical Presentations					
Proximal Muscle Weakness	5 (38.5%)	4 (14.3%)	2 (33.3%)	11 (23.4%)	0.20
Mechanic Hands	11 (84.6%)	5 (17.9%)	1 (16.7%)	17 (36.2%)	<0.001
mMRC 3	10 (76.9%)	6 (21.4%)	2 (33.3%)	18 (38.3%)	0.003
mMRC 4	2 (15.4%)	4 (14.3%)	0 (0.0%)	6 (12.8%)	0.60
Diagnoses					
Lupus	0 (0.0%)	1 (3.6%)	0 (0.0%)	1 (2.1%)	0.71
Seropositive Rheumatoid Arthritis	2 (15.4%)	1 (3.6%)	4 (66.7%)	7 (14.9%)	<0.001
Polymyositis	1 (7.7%)	0 (0.0%)	0 (0.0%)	1 (2.1%)	0.26
Myopathic Dermatomyositis	1 (7.7%)	1 (3.6%)	0 (0.0%)	2 (4.3%)	0.71
Limited Scleroderma	0 (0.0%)	1 (3.6%)	0 (0.0%)	1 (2.1%)	0.71
Diffuse Systemic Scleroderma	0 (0.0%)	2 (7.1%)	0 (0.0%)	2 (4.3%)	0.49
Mixed Connective Tissue Disease	2 (15.4%)	1 (3.6%)	2 (33.3%)	5 (10.6%)	0.081
Unspecified Inflammatory Arthritis	0 (0.0%)	1 (3.6%)	0 (0.0%)	1 (2.1%)	0.71
Undifferentiated CTD Overlap	0 (0.0%)	2 (7.1%)	0 (0.0%)	2 (4.3%)	0.49
Antisynthetase Syndrome	7 (53.8%)	11 (39.3%)	1 (16.7%)	19 (40.4%)	0.30
Amyopathic Dermatomyositis	1 (7.7%)	1 (3.6%)	0 (0.0%)	2 (4.3%)	0.71
CT-ILD	13 (100.0%)	21 (75.0%)	6 (100.0%)	40 (85.1%)	0.061
IPAF	0 (0.0%)	7 (25.0%)	0 (0.0%)	7 (14.9%)	0.061
Chest CT Diagnoses					0.005
COP	3 (23.1%)	1 (3.6%)	0 (0.0%)	4 (8.5%)	
DIP	0 (0.0%)	1 (3.6%)	0 (0.0%)	1 (2.1%)	
Indeterminate for UIP	0 (0.0%)	1 (3.6%)	0 (0.0%)	1 (2.1%)	
NSIP	3 (23.1%)	8 (28.6%)	1 (16.7%)	12 (25.5%)	
NSIP with OP Overlap	6 (46.2%)	15 (53.6%)	0 (0.0%)	21 (44.7%)	
Probable UIP	0 (0.0%)	1 (3.6%)	1 (16.7%)	2 (4.3%)	
UIP	1 (7.7%)	0 (0.0%)	3 (50.0%)	4 (8.5%)	
Other	0 (0.0%)	1 (3.6%)	1 (16.7%)	2 ( 4.3 %)	
Chest CT Diagnosed Severity					0.29
Mild	0 (0.0%)	6 (21.4%)	2 (33.3%)	8 (17.0%)	
Mild to Moderate	3 (23.1%)	6 (21.4%)	0 (0.0%)	9 (19.1%)	
Moderate	5 (38.5%)	12 (42.9%)	1 (16.7%)	18 (38.3%)	
Moderate to Severe	4 (30.8%)	3 (10.7%)	2 (33.3%)	9 (19.1%)	
Severe	1 (7.7%)	1 (3.6%)	1 (16.7%)	3 (6.4%)	
PFT Baseline Values					
FVC	1.92 (1.20, 2.45)	2.21 (1.13, 3.70)	2.34 (1.12, 3.59)	2.12 (1.12, 3.70)	0.44
FVC%	50.00 (41.00, 65.00)	66.00 (34.00, 125.00)	58.00 (44.00, 83.00)	59.00 (34.00, 125.00)	0.005
DLCO	8.60 (5.80, 16.70)	9.90 (3.40, 17.10)	13.10 (9.40, 20.60)	9.40 (3.40, 20.60)	0.11
DLCO%	34.00 (25.00, 57.00)	43.50 (16.00, 84.00)	46.00 (35.00, 71.00)	41.00 (16.00, 84.00)	0.28

Continuous variables summarized using median (range), and categorical variables summarized using *n* (%). Differences between therapy plans were evaluated using the Kruskal–Wallis Rank Sum test for continuous measures and the Pearson Chi-square test for proportions. All tests are two-sided and *p*-values less than 0.05 are considered statistically significant. mMRC: modified Medical Research Council scale for dyspnea.

**Table 2:** Summary of PFT outcomes.

	Baseline	6 Months	12 Months	Baseline to 6 Months	*p*-Value	6 Months to 12 Months	*p*-Value	Baseline to 12 Months	*p*-Value
FVC	2.120 (1.120, 3.700)	2.280 (1.120, 3.810)	2.310 (1.110, 3.720)	0.10 (−0.57, 1.10)	<0.001	−0.01 (−0.43, 0.52)	0.95	0.12 (−0.58, 1.03)	0.006
FVC%	59.000 (34.000, 125.000)	66.000 (38.000, 128.000)	65.500 (38.000, 123.000)	4.00 (−19.00, 34.00)	<0.001	0.00 (−12.00, 13.00)	0.63	4.50 (−19.00, 39.00)	0.002
DLCO	9.400 (3.400, 20.600)	10.600 (3.800, 19.300)	10.100 (2.040, 20.000)	0.60 (−5.10, 8.00)	0.052	0.55 (−15.56, 4.70)	0.017	1.05 (−10.56, 6.70)	0.001
DLCO%	41.000 (16.000, 84.000)	42.000 (20.000, 77.000)	44.000 (24.000, 80.000)	3.00 (−21.00, 38.00)	0.017	3.00 (−16.00, 16.00)	0.003	6.00 (−15.00, 25.00)	<0.001

Changes in PFT outcomes summarized with median (range). *p*-values represent evaluation of whether PFT change is significantly different over specified follow-up period using the Kruskal–Wallis Rank Sum test. All tests are two-sided and *p*-values less than 0.05 are considered statistically significant.

**Table 3:** Summary of treatment doses with PFT outcomes.

	Baseline to 6 Months		6 Months to 12 Months	
	*β* (95% CI)	*p*-Value	*β* (95% CI)	*p*-Value
FVC				
Rituximab 2 g increase	0.23 (−0.12, 0.57)	0.19	0.10 (−0.03, 0.22)	0.13
Mycophenolate 1 g increase	0.10 (−0.01, 0.21)	0.08	0.03 (−0.02, 0.09)	0.21
Prednisone 10 mg/d decrease	−0.07 (−0.12, 0.03)	<0.01	−0.06 (−0.10, 0.01)	0.01
FVC%				
Rituximab 2 g increase	4.97 (−5.92, 15.85)	0.36	2.95 (−0.69, 6.59)	0.11
Mycophenolate 1 g increase	2.17 (−1.39, 5.73)	0.23	1.09 (−0.46, 2.64)	0.16
Prednisone 10 mg/d decrease	−2.48 (−3.97, −0.98)	<0.01	−1.48 (−2.76, −0.19)	0.03
DLCO				
Rituximab 2 g increase	1.90 (−1.19, 5.00)	0.22	0.38 (−1.99, 2.75)	0.75
Mycophenolate 1 g increase	0.93 (0.04, 1.82)	0.04	0.93 (−0.02, 1.89)	0.06
Prednisone 10 mg/d decrease	−0.03 (−0.42, 0.36)	0.88	−0.14 (−0.91, 0.63)	0.71
DLCO%				
Rituximab 2 g increase	7.34 (−6.89, 21.57)	0.30	3.17 (−2.18, 8.51)	0.24
Mycophenolate 1 g increase	3.31 (−0.80, 7.43)	0.11	1.88 (−0.28, 4.04)	0.09
Prednisone 10 mg/d decrease	−0.77 (−2.51, 0.98)	0.38	−0.05 (−1.80, 1.69)	0.95

*β* represents the estimated average linear change in corresponding PFT outcome over specified study follow-up period for every 2 g increase in rituximab, 1 g increase in mycophenolate, and 10 mg/d decrease in prednisone. All models adjusted for age at baseline and sex. All tests are two-sided and *p*-values less than 0.05 are considered statistically significant.

**Table 4:** Summary of therapy plans with changes in PFT from baseline to 12 months.

	Median (Range)	*β* (95% CI)	*p*-Value
FVC			
Customized Therapy	0.31 (−0.17, 1.03)	ref	ref
Rituximab Only	0.03 (−0.21, 0.50)	−0.35 (−0.73, 0.03)	0.07
Mycophenolate Only	0.03 (−0.58, 0.91)	−0.15 (−0.38, 0.08)	0.21
FVC%			
Customized Therapy	9.00 (−6.00, 24.00)	ref	ref
Rituximab Only	2.00 (−4.00, 12.00)	−6.77 (−18.27, 4.72)	0.24
Mycophenolate Only	2.00 (−19.00, 39.00)	−1.48 (−8.50, 5.55)	0.67
DLCO			
Customized Therapy	1.85 (−1.40, 4.90)	ref	ref
Rituximab Only	−1.60 (−10.56, 0.10)	−7.21 (−10.33, −4.08)	<0.01
Mycophenolate Only	1.05 (−3.10, 6.70)	−0.84 (−2.66, 0.98)	0.36
DLCO%			
Customized Therapy	8.50 (−5.00, 23.00)	ref	ref
Rituximab Only	0.00 (−9.00, 19.00)	−11.28 (−23.21, 0.65)	0.06
Mycophenolate Only	6.00 (−15.00, 25.00)	−2.64 (−9.58, 4.30)	0.45

*β* represents the estimated average linear change in corresponding PFT outcome baseline to 12 months for patients on rituximab and mycophenolate monotherapy compared to patients on a customized MMF and RTX therapy plan. All models adjusted for age at baseline and sex. All tests are two-sided and *p*-values less than 0.05 are considered statistically significant.

**Table 5:** Summary of change in treatment dose from baseline to 6 months with PFT outcomes from 6 months to 12 months.

	*β* (95% CI)	*p*-Value
FVC		
Rituximab 2 g increase	0.15 (0.00, 0.30)	0.05
Mycophenolate 1 g increase	0.03 (−0.03, 0.10)	0.28
Prednisone 10 mg/d decrease	0.02 (−0.01, 0.05)	0.20
FVC%		
Rituximab 2 g increase	3.89 (−0.48, 8.26)	0.08
Mycophenolate 1 g increase	0.74 (−1.04, 2.52)	0.41
Prednisone 10 mg/d decrease	0.57 (−0.39, 1.54)	0.24
DLCO		
Rituximab 2 g increase	2.56 (−0.25, 5.36)	0.07
Mycophenolate 1 g increase	0.50 (−0.52, 1.52)	0.33
Prednisone 10 mg/d decrease	−0.05 (−0.61, 0.51)	0.86
DLCO%		
Rituximab 2 g increase	8.06 (2.12, 14.00)	0.01
Mycophenolate 1 g increase	1.18 (−0.98, 3.34)	0.28
Prednisone 10 mg/d decrease	−0.03 (−1.22, 1.16)	0.96

*β* represents the estimated average linear change in corresponding PFT outcome from 6-month to 12-month follow-up for every 2 g increase in rituximab, 1 g increase in mycophenolate, and 10 mg/d decrease in prednisone from baseline to 6 months. All models adjusted for age at baseline and sex. All tests are two-sided and *p*-values less than 0.05 are considered statistically significant.

## Data Availability

The original contributions presented in the study are included in the article. Further inquiries can be directed to the corresponding author.

## Supplementary Materials

The following supporting information can be downloaded at: https://www.mdpi.com/article/10.3390/diagnostics14232702/s1, Figure S1: Flowchart of study subject inclusion; Table S1. Interaction analysis of mycophenolate and rituximab dose; Table S2. Interaction analysis of decreasing prednisone (per 10 mg/d) and mycophenolate dose; Table S3. Interaction analysis of decreasing prednisone (per 10 mg/d) and rituximab dose; Table S4. Interaction analysis of increase in mycophenolate and change in rituximab from baseline to 6 months with PFT outcome changes from 6 months to 12 months; Table S5. Interaction analysis of decrease in prednisone with change in mycophenolate from baseline to 6 months with PFT outcome changes from 6 months to 12 months; Table S6. Interaction analysis of decrease in prednisone with change in rituximab from baseline to 6 months with PFT outcome changes from 6 months to 12 months.
