# Supplementary material for: Longitudinal Study of Patients with Connective Tissue Disease–Interstitial Lung Disease and Response to Mycophenolate Mofetil and Rituximab
Source: Diagnostics (Basel). 2024 Nov 30;14(23):2702. doi: 10.3390/diagnostics14232702 (PMC11640232; doi:10.3390/diagnostics14232702)
Supplement: Supplementary file 1 [file diagnostics-14-02702-s001.zip › diagnostics-3298697-supplementary.pdf]

Supplemental Figure S1: Flowchart of study subject inclusion.

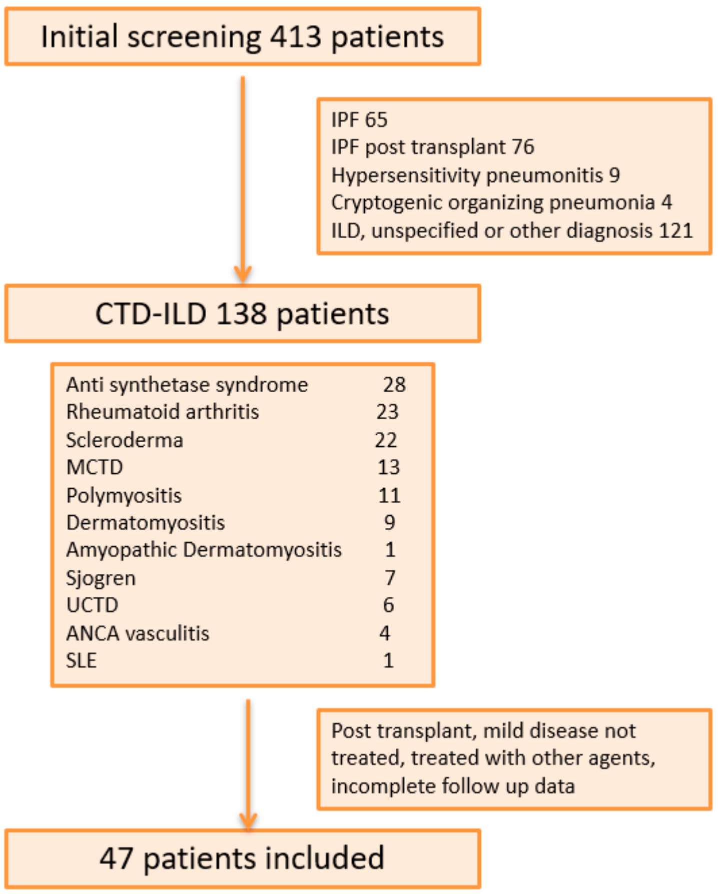

Supplemental Table S1. Interaction analysis of mycophenolate and rituximab dose.

	**Baseline to 6 Months**					**6 Months to 12 Months**				
	Rituximab not prescribed		Rituximab prescribed		Interaction	Rituximab not prescribed		Rituximab prescribed		Interaction
	β (95% CI)	P-value	β (95% CI)	P-value	P-value	β (95% CI)	P-value	βs(95% CI)	P-value	P-value
**FVC**	0.07 (-0.07, 0.20)	0.32	0.29 (0.09, 0.50)	**0.01**	0.24	0.08 (-0.02, 0.19)	0.12	0.02 (-0.04, 0.09)	0.45	0.32
**FVC%**	1.56 (-2.81, 5.93)	0.48	4.58 (-0.57, 9.73)	0.07	0.62	3.17 (-0.01, 6.35)	**0.05**	0.55 (-1.08, 2.19)	0.48	0.13
**DLCO**	0.69 (-0.20, 1.57)	0.12	1.84 (-3.33, 7.00)	0.34	0.40	0.00 (-0.85, 0.84)	0.99	1.35 (-0.41, 3.11)	0.12	0.16
**DLCO%**	2.39 (-1.80, 6.58)	0.26	5.75 (-15.22, 26.72)	0.45	0.60	**-0.72 (-4.55, 3.11)**	0.70	2.79 (0.61, 4.97)	**0.02**	0.10

β represents the estimated average linear change in corresponding PFT outcome over specified study follow-up period and dosage of rituximab for every 1 g increase in mycophenolate. All tests are two-sided and p-values less than 0.05 are considered statistically significant.

Supplemental Table S2. Interaction analysis of decreasing prednisone (per 10 mg/d) and mycophenolate dose.

	Baseline to 6 Months					6 Months to 12 Months				
	Mycophenolate not prescribed		Mycophenolate prescribed		Interaction	Mycophenolate not prescribed		Mycophenolate prescribed		Interaction
	β (95% CI)	P-value	β (95% CI)	P-value	P-value	β (95% CI)	P-value	β (95% CI)	P-value	P-value
**FVC**	0.00 (-0.15, 0.15)	1.00	-0.08 (-0.14, -0.03)	**<0.01**	0.36	0.05 (-0.33, 0.42)	0.78	-0.06 (-0.10, -0.02)	**0.01**	0.84
**FVC%**	0.40 (-3.07, 3.87)	0.79	-3.02 (-4.66,1.39)	**<0.01**	0.24	0.05 (-9.64, 9.73)	0.99	-1.47 (-2.79, -0.15)	**0.03**	0.93
**DLCO**	0.50 (-1.98, 2.99)	0.60	-0.12 (-0.52,0.29)	0.56	0.44	-3.41 (-12.08, 5.27)	0.37	-0.08 (-0.50, 0.35)	0.72	0.21
**DLCO%**	1.96 (-7.30, 11.22)	0.59	-1.23 (-3.02,0.57)	0.17	0.42	-0.30 (-9.98, 9.38)	0.94	-0.14 (-1.95, 1.67)	0.87	0.74

β represents the estimated average linear change in corresponding PFT outcome over specified study follow-up period and dosage of mycophenolate for every 10 mg/d **decrease** in prednisone. All tests are two-sided and p-values less than 0.05 are considered statistically significant.

Supplemental Table S3. Interaction analysis of decreasing prednisone (per 10 mg/d) and rituximab dose.

	Baseline to 6 Months					6 Months to 12 Months				
	Rituximab not prescribed		Rituximab prescribed		Interaction	Rituximab not prescribed		Rituximab prescribed		Interaction
	β (95% CI)	P-value	β (95% CI)	P-value	P-value	β (95% CI)	P-value	β (95% CI)	P-value	P-value
**FVC**	-0.07 (-0.12, -0.02)	**0.01**	-0.03 (0.34, -0.27)	0.79	0.76	-0.07 (-0.11, -0.02)	**0.01**	0.01 (-0.12, 0.15)	0.83	0.25
**FVC%**	-2.56 (-4.14, -0.98)	**<0.01**	-0.25 (-5.95, 5.45)	0.91	0.57	-1.62 (-3.13, -0.10)	**0.04**	-0.06 (-3.52, 3.40)	0.97	0.44
**DLCO**	0.00 (-0.39, 0.39)	0.99	-3.02 (-11.43, 5.40)	0.34	0.17	-0.11 (-0.51, 0.29)	0.58	-0.52 (-5.53, 4.50)	0.83	0.78
**DLCO%**	-0.70 (-2.44, 1.05)	0.42	-10.50 (-43.65, 22.65)	0.39	0.32	-0.29 (-2.10, 1.52)	0.74	2.10 (-4.98, 9.19)	0.53	0.46

β represents the estimated average linear change in corresponding PFT outcome over specified study follow-up period and dosage of rituximab for every 10 mg/d **decrease** in prednisone. All tests are two-sided and p-values less than 0.05 are considered statistically significant.

Supplemental Table S4. Interaction analysis of increase in mycophenolate and change in rituximab from baseline to 6 months with PFT outcome changes from 6 months to 12 months.

	PFT outcomes from 6 months to 12 months						
	Rituximab decreased		No change in Rituximab		Increase in Rituximab		Interaction
	β (95% CI)	P-value	β (95% CI)	P-value	β (95% CI)	P-value	P-value
**FVC**	NA (NA, NA)	NA	0.09 (0.01, 0.18)	**0.03**	-0.06 (-0.10, -0.02)	**0.01**	0.01
**FVC%**	NA (NA, NA)	NA	2.42 (0.00, 4.84)	**0.05**	-1.82 (-2.82, -0.81)	**<0.01**	0.01
**DLCO**	NA (NA, NA)	NA	0.79 (-0.60, 2.19)	0.25	0.15 (-0.91, 1.22)	0.74	0.51
**DLCO%**	NA (NA, NA)	NA	1.72 (-1.07, 4.51)	0.22	0.52 (-3.29, 4.33)	0.75	0.57

β represents the estimated average linear change in corresponding PFT outcome from 6 months to 12 months and change in dose of rituximab from baseline to 6 months for every 1 g increase in mycophenolate from baseline to 6 months. All tests are two-sided and p-values less than 0.05 are considered statistically significant.

Supplemental Table S5. Interaction analysis of decrease in prednisone with change in mycophenolate from baseline to 6 months with PFT outcome changes from 6 months to 12 months.

	PFT outcome change from 6 months to 12 months						
	Mycophenolate decreased		No change in Mycophenolate		Increase in Mycophenolate		Interaction
	β (95% CI)	P-value	β (95% CI)	P-value	β (95% CI)	P-value	P-value
**FVC**	0.05 (-0.04, 0.15)	0.09	0.01 (-0.03, 0.05)	0.58	0.03 (-0.06, 0.11)	0.49	0.23
**FVC%**	1.64 (0.07, 3.21)	0.05	0.15 (-1.06, 1.35)	0.81	1.07 (-0.79, 2.92)	0.22	0.35
**DLCO**	1.24 (-1.12, 3.59)	0.09	-0.10 (-0.91, 0.71)	0.80	-0.07 (-0.77, 0.63)	0.82	0.39
**DLCO%**	5.57 (-10.15, 21.29)	0.14	-0.09 (-1.54, 1.37)	0.90	-1.14 (-4.54, 2.27)	0.45	0.07

β represents the estimated average linear change in corresponding PFT outcome from 6 months to 12 months and change in dose of mycophenolate from baseline to 6 months for every 10 mg/d **decrease** in prednisone from baseline to 6 months. All tests are two-sided and p-values less than 0.05 are considered statistically significant.

Supplemental Table S6. Interaction analysis of decrease in prednisone with change in rituximab from baseline to 6 months with PFT outcome changes from 6 months to 12 months.

	**PFT outcome change from 6 Months to 12 Months**						
	Rituximab decreased		No change in Rituximab		Increase in Rituximab		Interaction
	β (95% CI)	P-value	β (95% CI)	P-value	β (95% CI)	P-value	P-value
**FVC**	NA (NA, NA)	NA	0.01 (-0.02, 0.05)	0.44	0.06 (0.01, 0.11)	**0.03**	0.41
**FVC%**	NA (NA, NA)	NA	0.41 (-0.67, 1.49)	0.44	1.69 (0.27, 3.10)	**0.03**	0.40
**DLCO**	NA (NA, NA)	NA	-0.08 (-0.69, 0.52)	0.78	0.12 (-1.13, 1.38)	0.82	0.82
**DLCO%**	NA (NA, NA)	NA	-0.31 (-1.52, 0.90)	0.60	0.92 (-3.50, 5.33)	0.63	0.53

β represents the estimated average linear change in corresponding PFT outcome from 6 months to 12 months and change in dose of rituximab from baseline to 6 months for every 10 mg/d **decrease** in prednisone from baseline to 6 months. All tests are two-sided and p-values less than 0.05 are considered statistically significant.
